# Palladium(0) Deposited on PAMAM Dendrimers as a Catalyst for C–C Cross Coupling Reactions

**DOI:** 10.3390/molecules16010427

**Published:** 2011-01-10

**Authors:** Tomasz Borkowski, Paweł Subik, Anna M. Trzeciak, Stanisław Wołowiec

**Affiliations:** 1 Faculty of Chemistry, University of Wrocław, 14 F. Joliot-Curie Str., 50-383 Wrocław, Poland; E-Mail: tomekb@eto.wchuwr.pl (T.B.); 2 Faculty of Chemistry, Rzeszów University of Technology, 6 Powstańców Warszawy Ave., 35-959 Rzeszów, Poland; E-Mail: eloppo@interia.pl (P.S.)

**Keywords:** palladium nanoparticles, dendrimers, Suzuki-Miyaura coupling, Heck coupling, Hiyama coupling, Sonogashira coupling, TEM

## Abstract

PAMAM dendrimers of generations G2–G3 as well as a partially substituted derivative of generation G4 and a low-molecular-weight tricyclic ligand **4** were used to bind Pd(0) nanoparticles. The obtained adducts were tested as catalysts for C–C cross-coupling reactions, such as the Suzuki-Miyaura, Hiyama, Heck and Sonogashira reaction. The highest yields of the coupling product, diphenylacetylene, were obtained with all the catalysts studied in the Sonogashira coupling performed in ethanol with K_2_CO_3_ as base. Very good results, 85–100%, were also found in the Suzuki-Miyaura cross-coupling, while the efficiency of the Hiyama coupling appeared lower, with 38–52% of 2-Methylbiphenyl formed. In all reactions, the G2–Pd(0) catalyst, containing an unmodified dendrimer, afforded the highest yields of the cross-coupling products.

## 1. Introduction

Dendritic macromolecules, well defined on a molecular level, are attractive supports for metals designed for different applications, for example in optoelectronics, magnetism, and catalysis [1,2,3,4,5,6]. The main advantage of dendrimers over polymers as supports for metal catalysts consists in their polydispersity, which is 1, and broad possibilities of modification of the surface with functional groups. As a result, in reaction with a metal source, monodisperse distribution of the metal can be achieved, which is very important for the properties of the resulting materials, such as solubility [7]. 

In particular, dendrimer-encapsulated metal nanoparticles (DENs) have found applications in catalysis. The most often studied dendrimeric systems are those based on polyamidoamine (PAMAM) dendrimers, which have been widely reviewed [6,8,9]. The catalytic properties of palladium supported on dendrimers (DSNs) have also been discussed in detail [10,11].

Palladium(0), platinum(0), and silver(0) have been shown to form nanocomposites upon binding to G3 (3.6 nm diameter) and G4 (4.5 nm diameter) PAMAM dendrimers [12]. The metal nanoparticles were formed upon addition of AgNO_3_, H_2_PtCl_4_, or Na_2_PdCl_4_ solutions followed by reduction with sodium borohydride. The nanoparticle sizes of the silver, platinum, and palladium were 5.6-7.5, 1.2-1.6, and 1.6-2.0 nm, respectively. Those nanocomposites catalyzed the reduction of 4-nitrophenol with sodium borohydride, the palladium(0) nanocomposite being the most active catalyst [12].

PAMAM dendrimers have been modified on the peripheries by functionalization, such as double phosphinomethylation of each terminal amine group of G0–G3 on silica support. After coordination of palladium(II), these materials were shown to be active catalysts for cyclocarbonylation of 2-allylphenols, 2-allylaniline, 2-vinylphenol, and 2-vinylaniline with CO/H_2_, affording five-, six- or seven-membered ring lactones and lactams [13]. 

A G4 PAMAM dendrimer modified by introducing 64 hydroxy groups on the surface (G4-OH) has been demonstrated by elegant NMR studies to encapsulate 55 palladium atom nanoparticles [14]. These nanocomposites are active catalysts for hydrogenation of allylic alcohols [15]. PAMAM-encapsulated Pd(0) nanoparticles and palladium bonded to phosphinoferrocenyl-terminated amidoamines catalyze the Heck reaction [16,17]. 

Similarly to PAMAM, poly(propylene imine) (PPI) dendrimers as well as their alkylated and arylated derivatives have been demonstrated to encapsulate palladium(0), especially in the presence of auxiliary diphenylphosphinobenzoic acid as the ligand coordinating Pd(0), which ionically anchors the metal inside the PPI dendrimer [18]. The dendritic Pd complexes efficiently catalyzed allylic amination of cinnamyl methyl carbonate with morpholine and the Heck reaction of iodobenzene with *n*-butyl acrylate [18]. Good results in the Heck reaction have also been obtained with the application of palladium complexes bonded to phosphine-modified poly(ether imine) dendrimers [19].

Several terminally modified PPI dendrimers have also been used to bind catalytic metals. Double phosphinomethylation of terminal amine groups of G1–G3 PPI followed by coordination of palladium(II) acetate gave a Pd(II) complex that was an active catalyst for the Sonogashira coupling [20]. A G1 poly(propylene imine) pyridylimine dendrimer has been converted into a PdCl_2_ complex in reaction with PdCl_2_(COD) [21]. After activation by methylaluminoxane (MAO) the metallodendrimer was used as a catalyst precursor for the polymerization of ethylene to obtain high-molecular-weight, high-density polyethylene [21] and then in the Heck reaction of iodobenzene with methyl acrylate, styrene and 1-octene [22]. Modified PPI dendrimers with nitrile terminal groups have been successfully used as supports to bind palladium(II) and copper(II) *in situ*, with the mixture successfully used in the Wacker oxidation of terminal alkenes with good selectivity towards methylketones [23]. The Stille reaction has been successfully performed in water with Pd DENs and Pd^II^-PAMAM complexes [24].

Dendrimers have also been used for the stabilization of Pd(0) nanoparticles in the Suzuki-Miyaura reaction [25,26]. Interestingly, G4 PAMAM-OH-supported Pd(0) nanoparticles have been observed to be more stable than Pd/PVP in this reaction [27,28]. Since the catalytic activity of Pd/PVP had been the focus of our recent studies [29,30], we decided to test Pd(0) nanoparticles bonded to phosphane-free G2, G3, and G4 PAMAM dendrimers with peripheral imidazolimine residues. Unmodified PAMAM dendrimers bind metal ions weakly and form slightly water-soluble adducts, while introducing Schiff bases as additional N or O-donors makes it possible to obtain more stable metal ion complexes due to the chelate effect. The catalytic activity of a palladium adduct with the tricyclic compound **4** [31], obtained in a similar way as those with PAMAM dendrimers, was also tested.

## 2. Results and Discussion

### 2.1. Synthesis and characterization of Pd(0) supported on G2, G3 and G4^25Ac30Im^ dendrimers

Metal(0) deposition on dendrimers usually exploits the coordination of metal ions originating from simple salts such as K_2_[PdCl_4_] [14], followed by reduction of the metal ion with borohydride, hydrogen, or hydrazine. Such procedures lead to monolayers of metals bound to surface functional groups, especially phosphines. On the other hand, PAMAM dendrimers are able to encapsulate metal nanoparticles, as studied in detail for G4-OH(Pd_55_) [14].

**Figure 1:**
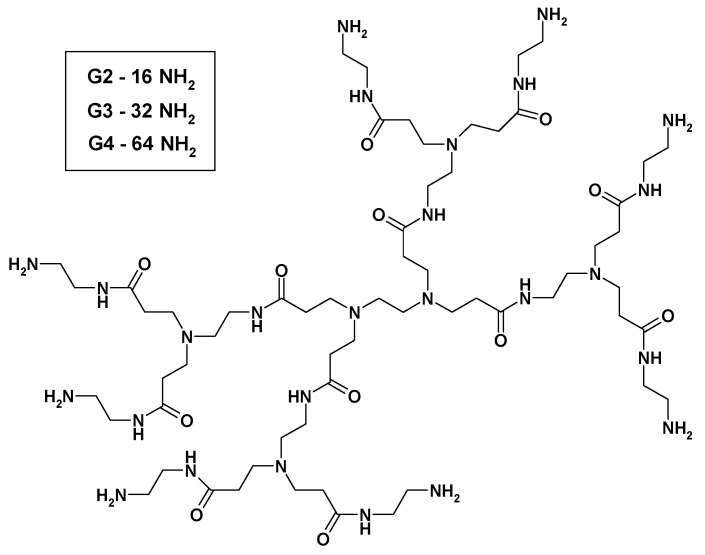
Chemical structure of PAMAM dendrimer G1.

**Figure 2:**
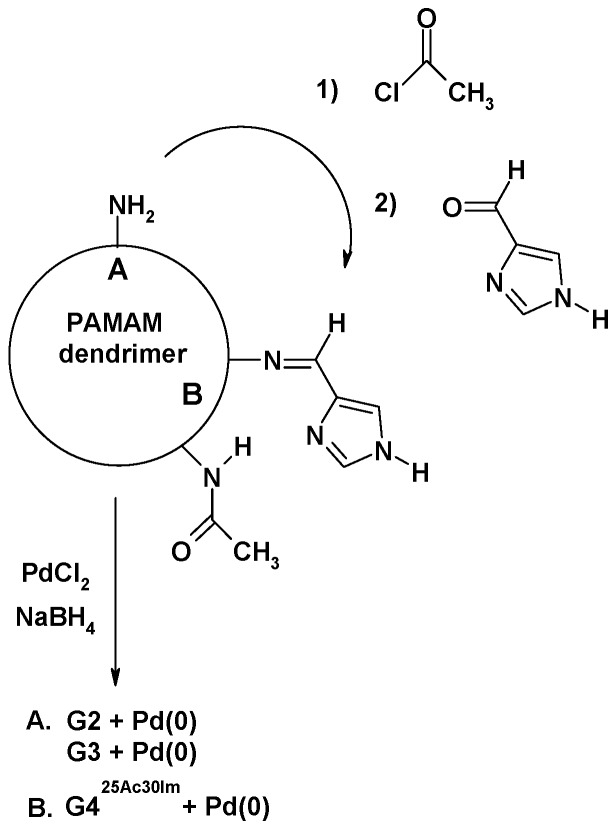
Schematic representation of synthesis of PAMAM dendrimer supported catalysts.

We applied a similar procedure to bind Pd(0) to G2 and G3 PAMAM dendrimers (Figure 1), except that methanol was used as a solvent and PdCl_2_ as a source of metal ions. The isolated yellow solid, containing Pd(II) presumably coordinated to surface amine groups was then dissolved again in methanol and reduced with sodium borohydride. The obtained adducts contained more than one Pd(0) per two amine groups (Figure 2, Table 1) and were slightly soluble in water. 

**Table 1:** The percentage of Pd(0) in adducts, number of Pd(0) equivalents per molecule (e) and number of Pd(0) atoms per one surface functional group (n).

Species	Number of surface groups	Pd (%)	e	n
**G2-Pd**	16 NH_2_	25	10	0.63
**G3-Pd**	32 NH_2_	27	24	0.75
**G4^25Ac30Im^-Pd**	19 Im; 29 NH_2_	24	60	1.25
**4-Pd**		19	2	1

**Figure 3:**
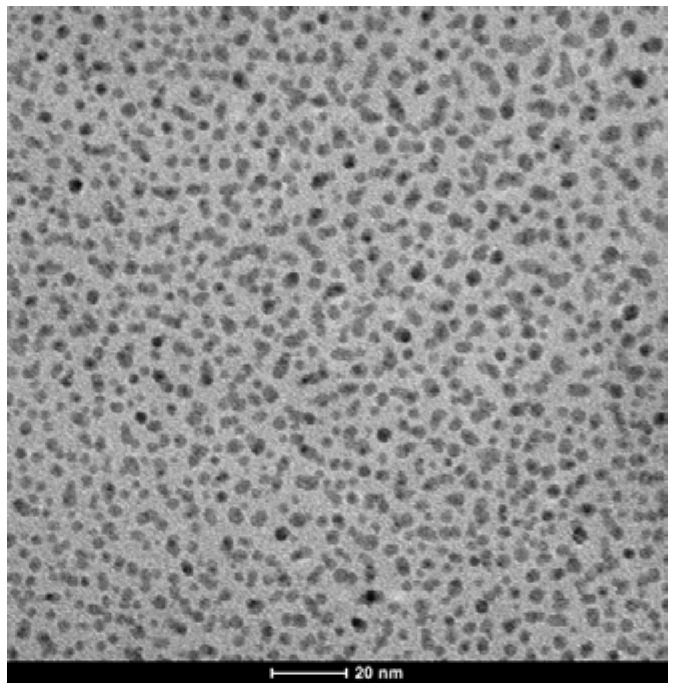
TEM micrographs of G2-Pd.

TEM examination of G2-Pd confirmed the presence of Pd(0) nanoparticles, uniformly distributed on the support. The size distribution of nanoparticles was quite narrow, with the center at 3.5 nm (Figure 3, Figure 5). Besides of small Pd(0) nanoparticles which dominated in TEM micrograph, very few bigger crystallites were also found.

**Figure 4:**
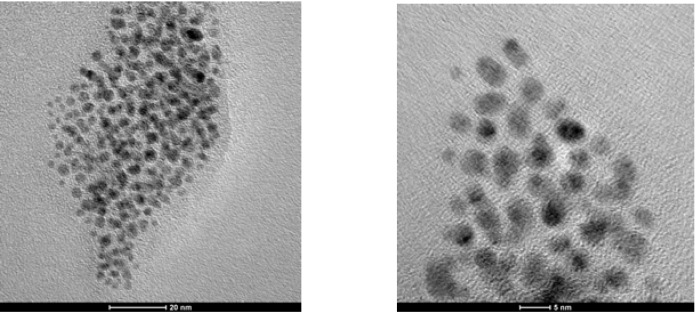
TEM micrographs of G3-Pd.

**Figure 5:**
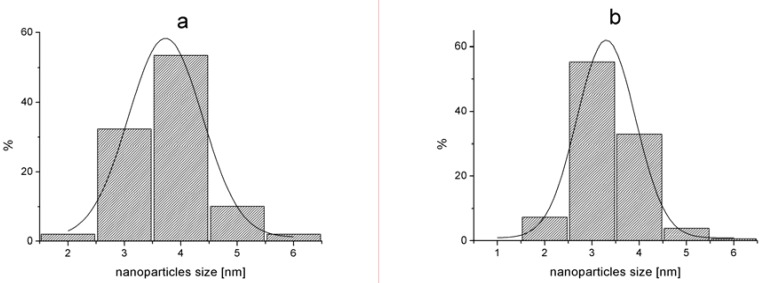
Pd(0) nanoparticles size analysis (a) G2-Pd, (b) G3-Pd.

TEM micrograph obtained for G3-Pd was different, as formation of bigger objects of 70–100 nm diameter containing small nanoparticles, was observed (Figure 4). The presence of palladium was confirmed by EDX, and the size analysis, based on *ca.* 180 particles, is presented in Figure 5. The average diameter of the nanoparticles was 3–4 nm, very close to the results obtained from XRD analysis (*ca.* 2 nm). The size of spheric nanoparticles corresponded to the diameter of G3 dendrimer suggesting that what was obtained was a monolayer of Pd(0) on G3.

**Figure 6:**
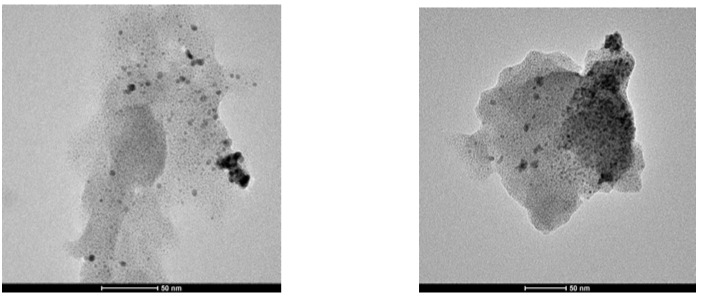
TEM micrograph of G4^25Ac30Im^-Pd.

The TEM picture of G4^25Ac30Im^-Pd shows the presence of Pd(0) nanoparticles of a rather different size, from 2–4 nm to aggregates of *ca.* 100 nm, not uniformly distributed on the surface (Figure 6).

**Figure 7:**
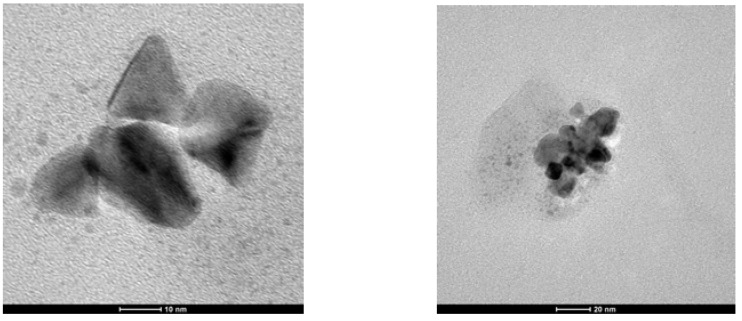
TEM micrograph of **4**-Pd(0).

The palladium content in **4**-Pd(0) was lower than in other catalysts studied; therefore, only scarce nanoparticles, not uniformly distributed, were observed in TEM. Interestingly, some of them presented well-defined geometrical shapes, triangles or squares (Figure 7).

### 2.2. Catalytic activity of Pd(0) supported on G2, G3 and G4^25Ac30Im^ dendrimers

Although numerous catalytic applications for Pd(0) on dendrimers have been presented, attempts with unmodified PAMAM dendrimers have, surprisingly, been unsuccessful [1,2,3,4,5]. Therefore, it was interesting to test the two adducts, G2-Pd and G3-Pd, in C–C cross-coupling reactions.

**Figure 8:**
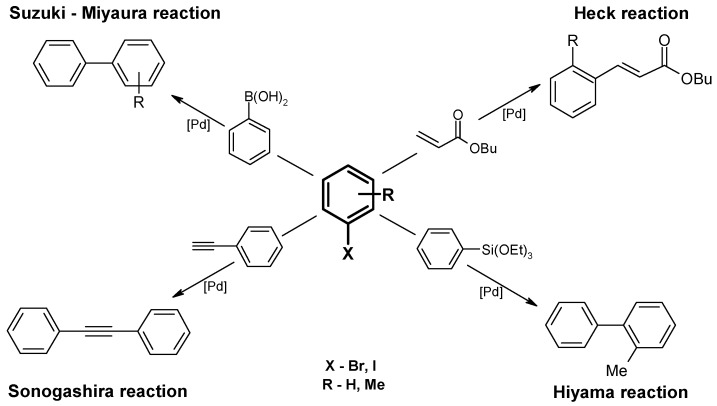
Scheme of studied C-C cross-coupling reactions.

Catalytic activity of the obtained Pd(0) catalysts was tested in the reactions shown in Figure 8. First, the Suzuki-Miyaura reaction was performed at 80 °C with fairly good results represented by 85–100% yield of 2-Methylbiphenyl (Table 2). The G2-Pd catalyst exhibited the highest productivity and the obtained results were comparable to those found for the Pd(0)/PVP nanocatalyst [30]. 

**Table 2:** The yield^a^ of products obtained in C-C cross-coupling reactions catalyzed by G2-Pd, G3-Pd, G4^25Ac30Im^ –Pd and **4**-Pd.

Reaction Catalyst	Suzuki-Miyaura	Hiyama	Heck				Sonogashira	
	2-propanol -water Cs_2_CO_3_80 °C, 3 h	ethylene glycol NaHCO_3_80 °C, 3 h	DMF Cs_2_CO_3_ 120 °C, 4 h	DMF Cs_2_CO_3_ 140 °C, 4 h	DMF Cs_2_CO_3 _140 °C, 4 h [Bu_4_N]Br 3 × 10^-4^ mol	DMF Cs_2_CO_3_ 140°C, 4 h[Bu_4_N]Br 6 × 10^-4^ mol	2-propanol Et_3_N 80 °C, 3 h	ethanol K_2_CO_3_ 80 °C, 3 h
G2-Pd	100	52	5	74	84	92	2	93
G3-Pd	98	32	0	0	3	0	0	87
G4^25Ac30Im^ -Pd	85	35	0	4	14	15	0	93
4- Pd	4 (5^b^; 17^c^)	8	-	0	28	26	-	95

^a^ conversion to the coupled product determined by GC; ^b ^4-bromotoluene was used as a substrate; ^c^ bromo-benzene was used as a substrate.

Next, recycling attempts were undertaken with Pd(0) catalysts separated from the reaction mixture by decantation. A remarkable decrease in product yield was observed in all cases as early as the second run and an average yield as low as *ca.* 20% was noted in the third run. A similar decrease of the catalytic activity of PAMAM stabilized Pd(0) nanoparticles in Suzuki-Miyaura reaction was previously reported [32]. The decrease in catalytic activity can to some extent be explained by agglomeration of Pd(0) nanoparticles, as was confirmed by TEM measurements; however, simple loss of material during catalyst recovery should also be considered.

Interestingly, much better results in recycling have been obtained when Pd immobilized on PAMAM was captured in a microporous network polymer [25]. Another successful method of stabilization of Pd bonded to PAMAM was its planting in SBA-15 [26]. The catalyst prepared in this way shown outstanding activity and recyclability in microwave assisted Suzuki-Miyaura coupling reaction [26].

**Figure 9:**
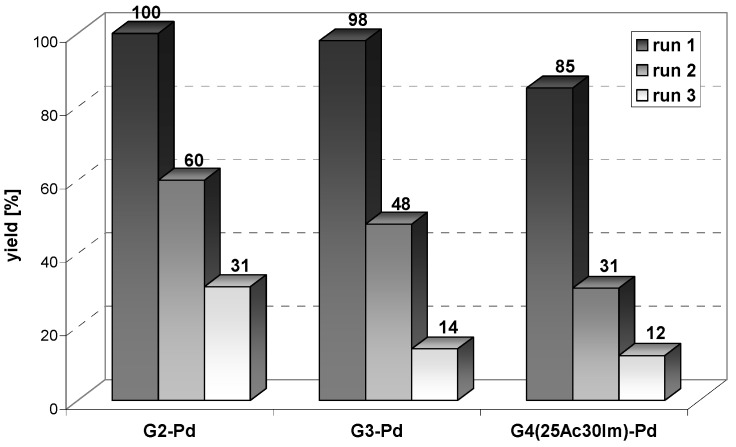
Results of recycling of G2-Pd, G3-Pd and G4^25Ac30Im^ –Pd in Suzuki-Miyaura reaction.

The next reaction to be tested was the Hiyama coupling, which facilitates obtaining the same cross-coupling product as the Suzuki-Miyaura reaction, namely 2-Methylbiphenyl. Under similar conditions, the yield of the Hiyama reaction was lower, ranging from 52% for G2-Pd to 32% for G4^25Ac30Im^ -Pd. Again, G2-Pd was the most active (Table 2).

In a model Heck reaction of bromobenzene and butyl acrylate, no products were formed at 120 °C, whereas at 140 °C 74% of butyl cinnamate was formed with G2-Pd catalyst. Less than 10% of the product was found with the remaining catalysts under study. To check whether the yield of the Heck reaction can be improved by introduction of [Bu_4_N]Br, the next two series of experiments were performed in the presence of [Bu_4_N]Br in amounts of 3 × 10^-4^ mol and 6 × 10^-4 ^mol. A positive influence of [Bu_4_N]Br (or other tetraalkylammonium salts) was expected on the basis of previous results reported for Heck reactions catalyzed by Pd(0)/PVP [29], Pd/Al_2_O_3_[33] and Pd(OAc)_2_ [34]. In reactions with dendrimer-supported palladium, an increase in yield after introduction of [Bu_4_N]Br was found for G2-Pd and G4^25Ac30Im^-Pd. Surprisingly, G3-Pd was practically not influenced by the salt. Also, in the case of G4^25Ac30Im^-Pd, only 14–15% of the product was formed, while in other systems, both homogeneous and heterogeneous, much better results were obtained under similar conditions [29,30,31,33,34].

Pd(0) deposited on PAMAM dendrimers did not catalyze the Sonogashira coupling; only 2% of the product, diphenylacetylene, was formed in the presence of G2-Pd when 2-propanol was used as a solvent and Et_3_N as a base. However, a change of both the solvent and the base, using ethanol and K_2_CO_3_, resulted in an increase in the yield of diphenylacetylene to 87–94% (Table 2).

It had been demonstrated previously that the catalytic activity of metal deposited on dendrimers decreased with the generation number [1]. This was also the case in the present study, although in other systems an increase of the catalytic activity with increasing generation of the dendrimer was noted. Such positive dendritic effect was probably caused by more stable coordination of palladium to the dense amino groups inside the dendrimer [18]. 

In the next step of our studies, we modified the G4 dendrimer by partly blocking the amine surface groups in order to dilute the metal centers on the surface of the dendrimer and introduced an additional N-donor by binding the imidazolyl moiety to provide a more stable metal binding site. The acetylation of 16 amine groups of the 64 available in the starting G4, followed derivatization of 19 other amine groups with 4(5)-imidazolecarboxyaldehyde, leaving another 29 amine groups on the surface, resulted in the formation of G4^25Ac30Im^. This species was able to bind as many as 1.6 Pd(0) atoms per nitrogen donor. The high percentage of Pd(0) evidenced that besides the layering, the metal was also encapsulated, or layering of metal nanoparticles took place. However, as can be deduced from the data presented in Table 2, the modifications performed did not lead to clear advantages in catalytic reactions. G4^25Ac30Im^-Pd showed a lower catalytic activity than G2-Pd in all the reactions studied. However, G4^25Ac30Im^-Pd appeared to be a better catalyst than G3-Pd in the Heck and Sonogashira reactions.

**Figure 10:**
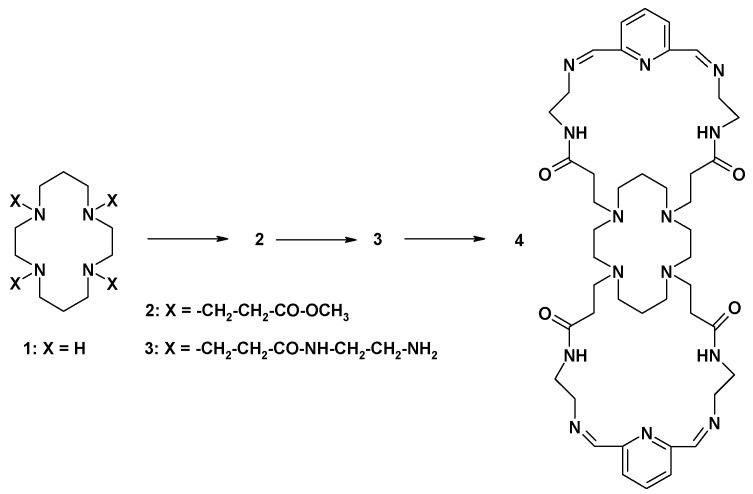
The synthetic route to compound **4**.

Another compound tried in catalytic tests was an adduct between the tricyclic compound **4** and Pd(0) obtained in similar way as those with PAMAM dendrimers (Figure 10). The neutral ligand **4** has recently been demonstrated to bind two manganese(II) ions within Schiff-base loops [31] upon reaction of **4** with MnCl_2_ in methanol, while under the same conditions copper(II) chloride formed the adducts (CuCl_2_)_n_ with **4**, where n = 5, 6, and 7 were detected by mass spectral assignment. Therefore, **4 **was tried here as a stabilizer for Pd(0) nanoparticles and gave very a promising result: 95% yield in the Sonogashira reaction. The same catalyst, **4**-Pd, also exhibited medium activity in the Heck cross-coupling in the presence of [Bu_4_N]Br, forming 26–28% of butyl cinnamate. In contrast to Pd(0) deposited on PAMAM dendrimers, **4**-Pd is a rather poor catalyst for the Suzuki-Miyaura and Hiyama reactions (Table 2).

### 2.3. Analysis of G3-Pd after Suzuki-Miyaura reaction

**Figure 11:**
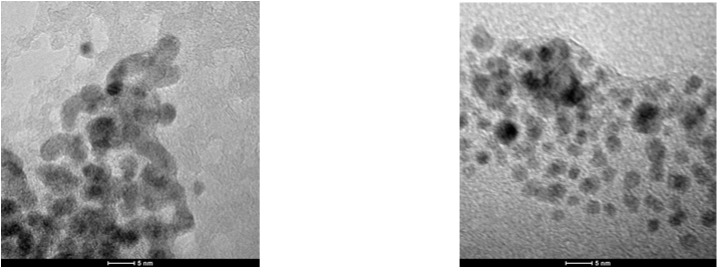
TEM micrograph of G3-Pd isolated after Suzuki-Miyaura reaction.

The G3-Pd catalyst was isolated from the reaction mixture after the Suzuki-Miyaura reaction and analyzed using XRD and TEM methods to get information about their eventual structural changes. Both methods confirmed the aggregation of Pd(0) nanoparticles and the formation of big objects, up to 100 nm. According to XRD, the average size of nanoparticles increased from 1.8 nm to 5.9 nm. Similarly, the average diameter of Pd(0) nanoparticles in TEM analysis was *ca.* 4.5 nm, although it should be pointed out that aggregates were not included in this statistic (Figure 11).

The palladium content estimated by the ICP method in the G3-Pd catalyst used was 25.7% versus 27.4% before the reaction. Thus, only *ca.* 6% of palladium was leached from the dendrimer. However, there is no strong evidence that palladium bonded to the dendrimer presents the main catalytically active form and the high catalytic activity of the leached palladium should be also considered.

## 3. Experimental

### 3.1. Physical measurements

The ^1^H- and ^13^C-NMR spectra were recorded with a Bruker 500 MHz UltraShield^TM^Plus instrument. For the spectral assignments the standard 1-D and 2-D COSY, NOESY, HSQC and HMBC measurements were performed. 

TEM measurements were performed using a FEI Tecnai G^2^ 20 X-TWIN electron microscope operating at 200 kV and providing 0.25 nm resolution. Specimens for TEM studies were prepared by putting a droplet of a colloidal suspension on a copper microscope grid covered with a perforated carbon film followed by evaporating the solvent under IR lamp for 15 minutes. The mean particle diameter and size distributions were calculated by counting at least 180 particles from the enlarged micrographs. 

X-ray powder diffraction (XRD) measurements were performed using DRON-3 diffractometer (Ni-filtered Cu-K_α_ radiation); a scan rate of 0.5 deg/min was used to record the patterns in the 2θ range of 20–80°. 

Palladium content was estimated by the ICP method, after mineralization of a weighted sample with 4.5 cm^3^ of HCl (35–38%) and 1.5 cm^3^ of HNO_3_ (65%). The mixture was refluxed for 4 h, colled down, and diluted next with H_2_O to 25 cm^3^. 

### 3.2. Syntheses

PAMAM dendrimers of generations **G2**, **G3** and **G4** on an ethylenediamine core were synthesized according to the published procedure [35]. The derivative of the **G4** dendrimer as Schiff base obtained from 4(5)-imidazolecarboxyaldehyde was obtained as follows: ***G4^25Ac30Im^***: The derivative of the **G4 **dendrimer with amine groups partially blocked with acetyl substituent (25% of amine groups on average), **G4^25Ac^**, was obtained in reaction of **G4** with acetyl chloride as described previously [36]. This was further converted by reaction with 4(5)-imidazolecarboxyaldehyde to derivatize 30% of all amine groups. The resulting modified dendrimer **G4^25Ac30Im^** was obtained with the 95% yield. The compound was characterized by ^1^H-NMR spectroscopy as reported in [36].

*1,11:4,8-bis(pyridin-2,6-diyl-bis(2-(N-(2-formidoylethylene)carbamoyl)ethylene))-1,4,8,11-tetra-azacyclotetradecane(**4**)and palladium(0) adduct(**4-**Pd)*: **4** (MW = 855 g/mol) was obtained from cyclam (**1** in Scheme 1) by consecutive addition of methyl acrylate, followed by condensation with ethylenediamine and further reaction with pyridine-2,6-dicarbaldehyde as described in [31]. The Pd(0) adduct was obtained as follows: Pd(II) chloride (0.125 g, 0.70 mmol) dissolved in methanol (300 mL) was added dropwise to a warm chloroform solution of **4** (0.300 g, 0.35 mmol). The mixture was refluxed for 5 h. A brown solution was formed, to which sodium borohydride solution in methanol was added in large excess. A black solid was formed, which was filtered off and washed with methanol to remove excess of sodium borohydride and other impurities. A **4-**Pd adduct was obtained, in which two Pd(0) metal atoms were bound to macrocycle **4** as determined by ICP. 

*Synthesis of palladium(0) adducts with PAMAM dendrimers and their derivatives*: Palladium(0) adducts with **G2** (MW = 3,256 g/mol), **G3 **(MW = 6,909 g/mol), and **G4^25Ac30Im^** (MW = 16,371 g/mol) were obtained by addition of excess palladium dichloride to the dendrimers (both in methanol), followed by reduction of the isolated PAMAM-PdCl_2_ complexes with sodium borohydride as follows:

Palladium(II) chloride 0.2943g (1.66 mmol) was dissolved in methanol (600 mL). **G2** (0.2815 g, 1.38 mmol of NH_2_) or **G3** (0.1914 g, 0.886 mmol of NH_2_) or **G4^25Ac30Im^** (0.1016 g, 0.0061mmol) in methanol (100 mL) were added dropwise to the methanol solution of PdCl_2_. The reaction mixtures were refluxed for 5 hours. Dark yellow precipitates were formed upon reconcentration of the methanolic solutions, which were filtered off and washed with methanol. The isolated palladium(II) adducts were then dissolved in methanol and reduced with freshly prepared sodium borohydride solution in methanol. Dark brown to black precipitates were formed, which were washed with methanol to remove excess of the reducing agent. The palladium content was determined by IPC technique and the percentage of palladium(0) as well as the number of equivalents per amine group were calculated (Table 1).

### 3.3. Catalytic reactions

The Suzuki-Miyaura, Hiyama, Heck and Sonogashira reactions were carried out in a Schlenk tube under N_2_ atmosphere, with magnetic stirring. Reagents for Suzuki-Miyaura reaction: phenylboronic acid (0.135 g, 1.1 mmol), 2-bromotoluene (0.118 mL, 1 mmol), Cs_2_CO_3_ (0.652 g, 2 mmol), 2-propanol (2.5 mL) and water (2.5 mL), palladium catalyst (1 × 10^-5^ mol of palladium). Reagents for Hiyama reaction: triethoxy(phenyl)silane (0.26 cm^3^, 1.1 mmol), 2-bromotoluene (0.118 mL, 1 mmol), NaHCO_3_ (0.168 g, 2 mmol), ethylene glycol (5 mL), palladium catalyst (1 × 10^-5^ mol of palladium). Reagents for Heck reaction: 2-bromotoluene (0.118 mL, 1 mmol) or bromobenzene (0.105 mL, 1 mmol), butyl acrylate (0.157 mL, 1.1 mmol), NaHCO_3_ (0.168 g, 2 mmol) or Cs_2_CO_3_ (0.652 g, 2 mmol), DMF (5 mL), palladium catalyst (1 × 10^-5^ mol of palladium), eventually [Bu_4_N]Br (0.097 g, 3 × 10^-4^ mol or 0.193 g, 6 × 10^-4^ mol). Reagents for Sonogashira reaction: iodobenzene (0.110 mL, 1 mmol), phenylacetylene (0.110 mL, 1 mmol), K_2_CO_3_ (0.276 g, 2 mmol), 2-propanol or ethanol (5 mL). Reagents were introduced directly to the Schlenk tube. Next, the Schlenk tube was sealed with a rubber tap and introduced into an oil bath pre-heated to 80 °C for Suzuki-Miyaura, Hiyama and Sonogashira reactions or to 120 °C–140 °C for Heck reaction. The reactions were carried out at 80 °C or 140 °C for 3 or 4 hours and after this time cooled down. Organic products were separated by extraction with hexane (4 mL, 3 mL and 3 mL). The combined extracts (10 mL) were analyzed by GC-FID (Hewlett Packard 8452A) with 0.076 mL of dodecane as an internal standard to determine the yield of products. GC-MS (Hewlett Packard 8452A) was used for product identification.

## 4. Conclusions

We have demonstrated that Pd(0) nanoparticles supported on PAMAM dendrimers can be used as suitable catalysts in four important C–C cross-coupling reactions, namely the Suzuki-Miyaura, Hiyama, Heck, and Sonogashira reaction. In all cases the best results were obtained with a G2-Pd catalyst containing an unmodified PAMAM dendrimer. In the Suzuki-Miyaura reaction, high yields of 2-Methylbiphenyl were obtained with G2-Pd, G3-Pd, and G4^25Ac30Im^-Pd, whereas very low activity was exhibited by Pd(0) bonded to the tricyclic ligand **4**. In recycling experiments, a decrease in yield was noted, which can be explained by agglomeration of Pd(0) nanoparticles, as confirmed by XRD and TEM for G3-Pd. Alternatively, the leaching of palladium was considered as the cause of the activity decrease; however, according to the ICP analysis of the catalyst isolated from the reaction mixture, only *ca.* 6% of palladium was lost from the dendrimer during recycling. On the basis of the literature reports one can expect that PAMAM bonded Pd(0) nanoparticles can be additionally stabilized by attachment to mezoporous or microporous materials [25,26]. The efficiency of the Hiyama reaction in the formation of 2-Methylbiphenyl was lower than that of the Suzuki-Miyaura for all catalysts.

The Heck coupling of bromobenzene with butyl acrylate can be performed at 140 °C, because only traces of product were formed at lower temperatures. With the G2-Pd catalyst, a 74% yield was obtained after 4 h, which is a better result than obtained testing the Pd/PVP catalyst [29]. Interestingly, the addition of [Bu_4_N]Br, which can function as a phase-transfer agent or stabilizer of Pd(0) nanoparticles [37,38], resulted in an increase in yield in reaction with G2-Pd, G4^25Ac30Im^-Pd, and **4**-Pd. In contrast, G3-Pd was practically not affected by the presence of [Bu_4_N]Br. Considering our previous results, which showed that the presence of [Bu_4_N]Br facilitated the formation of soluble Pd(II) species [29], one can conclude that such a process is not observed for G3-Pd. Similarly, a relatively small increase in the yield of the cross-coupling product after introduction of [Bu_4_N]Br in reactions catalyzed by G4^25Ac30Im^-Pd can indicate a rather limited participation of soluble palladium forms in the catalytic process. However, more investigations are needed to gain a better understanding of the nature of the systems studied.

The Sonogashira cross-coupling, leading to phenylacetylene, was very successfully performed with all the catalysts studied, including **4**-Pd. It should be pointed out that this reaction is very sensitive to the conditions applied, namely the kind of solvent and base used.
